# Editorial: Stress and Immunity

**DOI:** 10.3389/fimmu.2019.00245

**Published:** 2019-02-14

**Authors:** Yong-Soo Bae, Eui-Cheol Shin, Yoe-Sik Bae, Willem Van Eden

**Affiliations:** ^1^Department of Biological Science at College of Natural Science, Sungkyunkwan University, Suwon, South Korea; ^2^School of Medical Science and Engineering, Korea Advanced Institute of Science and Technology, Daejeon, South Korea; ^3^Infectious Diseases and Immunology, Utrecht University, Utrecht, Netherlands

**Keywords:** stress protein, cell stress, psychological stress, heat shock (stress) proteins, unfolded protein response (UPR)

Research regarding stress and its relation to the immune system has always been of interest for the medical and scientific community. In relation to the “Stress and Immunity” Research Topic we have to recognize the existence of two distant and seemingly unrelated forms of stress: physicochemical stress and psychological stress. In both forms of stress we see that the body has a tenacious ability to adapt to the changes in the environment thus adjusting itself accordingly. But the interplay between different stressors and the ever-elusive immune system has made this research increasingly fascinating.

Both forms of stress are worth studying since both have a bearing on the overall health status of an individual. Physicochemical stress results from environmental factors such as food/nutrition, toxins, metabolic disorders, infections, and inflammation. It is known that if the immune system is compromised and cannot properly cope with physicochemical stresses, or if the stress exceeds the regular adaptability of the immune system, this may lead to disease states or fatal conditions. On the other hand, we are all well aware of the effects of psychological stress on the body and the immune system altogether. Long-term and chronic stress leads to persistently high cortisol and corticosteroid levels, which cause resistance to cortisol and impaired anti-inflammatory effects on the immune system. Such effects result in chronic infection, chronic inflammatory autoimmune diseases, or cancers as well as other physiological disorders. Chronic stress also inhibits the cross talk of immune cells and signaling networks.

A relatively large proportion of the contributions in this Research Topic are dealing with the effects of stress in tumor models. It is interesting to note in this era of rising interest in cancer immunotherapies, that both of the aforementioned forms of stress are associated with malignancies and are found to have an impact on tumor immunity and the efficacy of tumor immunotherapies. The role of antidepressants in a murine tumor model was reported by Di Rosso et al. in the pursuit of finding a relationship between psychological-chronic stress and its effects on immune homeostasis and consequently tumor biology. Both of the antidepressants (i.e., fluoxetine and sertraline) were selective serotonin reuptake inhibitors (SSRIs). In this study, authors reported a reciprocal relationship between chronic stress and antitumor immunity resulting in tumor growth and metastasis. Although one may consider there may have been direct effects of the drugs on tumor biology, the inhibition of psychological stress by these drugs appears to restore antitumor immunity.

Another aspect worth exploring is the effect of stress-induced sympathetic adrenergic signaling on the immune system, since adrenergic signaling has been shown to inhibit immune responses in both autoimmune diseases and infection models. Qiao et al. discussed such stress-mediated adrenergic effects on antitumor immunity. They discovered that reduction in adrenergic stress by use of β-blockers reverses immunosuppression and significantly improves responses to checkpoint inhibitor immunotherapy. In the case of tumor models, the negative effects of psychological stress on immune performance are seen in a similar manner as consequences of physicochemical stress.

The role of hypoxic stress in the tumor microenvironment is another important facet of untoward anti-tumor immunity. Terry et al. reviewed the many ways in which hypoxic stress can lead to resistance to cell-mediated cytotoxicity and consequently to immune suppression. Talking about hypoxia as a stressor, Ostheimer et al. have introduced osteopontin (OPN) as a new tumor hypoxia-related marker. In patients with non-small-cell lung cancer, serum levels of stress protein HSP70 were found to correlate with OPN and increased levels of these markers were associated with decreased overall survival.

Although the Ostheimer at al. did not discuss the possible immune mechanisms of these correlations, it may be attractive to assume a possible relationship with the issues discussed by Barbera Betancourt et al. In the paper of Barbera Betancourt et al. stress proteins such as HSP70 play a distinctive role in the induction of immune suppressive regulatory T cells. While the evidence for this is obtained from extensive studies in models of chronic autoimmune arthritis, it is likely that similar mechanisms are active in the tumor microenvironment. Alternative and less immunosuppressive effects of stress on cells of the immune system are discussed by Antonangeli et al. Various types of stress are mentioned as triggers for the modulation of NKG2D ligands. Such ligands are recognized by activating NKG2D receptors on, for example, natural killer cells, which through the activation via these receptors can clear stressed mucosal epithelial cells.

In the paper by Yang et al. C/EBP homologous protein (CHOP) is discussed as a main factor in endoplasmatic reticulum stress (ER stress) induced apoptosis and its resulting implication in human diseases, such as cancer, diabetes, neurodegeneration, and fibrosis. CHOP is, besides GRP78 and Atf4, also one of the ER stress proteins studied by Gan et al. The upstream reduction of these proteins was seen in the process of leptin-mediated inhibition of autophagy. Such leptin-mediated autophagy inhibition may offer a potential new means for the therapy of metabolic syndrome in mammals.

The relationship between stress and the immune system is bidirectional and can affect each other in ways that are quite elusive and thus has attracted much attention from the scientific community. The team of Holzer et al. has introduced the reverse path of immune function leading to stress responses, especially from the visceral system to brain function, behavior, and stress coping. Effects of immune activation stress on brain function may well have a bearing on mental health and may lead to novel therapeutic possibilities.

Finally, nutrient deprivation as a stressor for bacterial species and as an adaptation of the fish immune system to limit bacterial invasion was described by Martínez et al. In the notothenoid sub-Antarctic fish model, it was found that bacterial *Piscirickettsia salmonis* infections were reduced by modulation of iron metabolism in the liver and brain.

Altogether, after having seen all individual contributions to this Research Topic, we see that the topic of “Stress and Immunity” is broad and has its roots in an active research field that is fundamental to the biology of health and disease. The far-reaching implications of stress-related immune functioning make this an intriguing subject important for future studies to gain better understanding.

## Author Contributions

WV and Yong-SooB wrote the paper together. All authors listed have made a substantial, direct and intellectual contribution to the work, and approved it for publication.

### Conflict of Interest Statement

The authors declare that the research was conducted in the absence of any commercial or financial relationships that could be construed as a potential conflict of interest.

